# Pancake kidney in infant: A case report with literature review

**DOI:** 10.1016/j.radcr.2025.10.054

**Published:** 2025-11-15

**Authors:** Rawa Bapir, Wriya N. Sabr, Soran H. Tahir, Bnar Sardar Saida, Bilal A. Abdullah, Karzan M. Hasan, Honar Othman Kareem, Hawkar A. Nasralla, Berun A. Abdalla, Nali H. Hama, Fahmi H. Kakamad

**Affiliations:** a Scientific Affairs Department, Smart Health Tower, Madam Mitterrand Street, Sulaymaniyah 46001, Iraq; b Kscien Organization for Scientific Research (Middle East office), Azadi Mall, Hamdi Street, Sulaymaniyah 46001, Iraq; c Department of Urology, Sulaymaniyah Surgical Teaching Hospital, Sulaymaniyah 46001, Iraq; d College of Medicine, University of Sulaimani, Madam Mitterrand Street, Sulaymaniyah 46001, Iraq; e Nephrology Department, Shar Teaching Hospital, Malik Mahmood Ring Road, Sulaymaniyah 46001, Iraq; f Dr. Jamal Ahmad Rashid's Pediatric Teaching Hospital, Qanat Street, Sulaymaniyah 46001, Iraq

**Keywords:** Fused kidneys, Ureteropelvic junction obstruction, Pyeloplasty, Pelvic kidney, Ectopic kidney

## Abstract

Pancake kidney (PK) is a rare anomaly, accounting for only 2% of all cases of fused kidneys. In this condition, the renal pelvis faces anteriorly, the ureters do not cross, and the collecting systems of the kidneys are separate, with no communication between the 2 sides. The PK may be associated with various urinary tract defects and anomalies in other organs. This report presents a case of an infant diagnosed with pelvic PK accompanied by ureteropelvic junction (UPJ) obstruction and an undescended right testis, representing an exceedingly rare combination of conditions. A 2-month-old male infant presented with an absent right testis. Ultrasound identified the right testis in the inguinal canal and revealed pelvic kidneys with malrotation. The right kidney showed mild hydronephrosis, while the left exhibited moderate hydronephrosis consistent with left UPJ obstruction and PK. A retrograde pyelogram confirmed mild UPJ stenosis, prompting the placement of a left JJ stent. The infant underwent right-sided orchidopexy and was scheduled for 6-month follow-ups. A review of 10 reported PK cases from the past decade revealed that 7 patients (70%) were male, and 3 (30%) were female, indicating a male predominance. The age at diagnosis ranged from 12 to 90 years. The retroperitoneal space was the site of PK in 2 cases (16.7%), with 1 case coexisting with rectosigmoid carcinoma. The PK may coexist with conditions such as UPJ obstruction and undescended testes. If asymptomatic, it can be managed conservatively, with monitoring to prevent complications.

## Background

Fetal kidneys first appear as small buds located near the urinary bladder. As development progresses, they migrate upward to take a lumbar position and become retroperitoneal. In rare cases, one right or left kidney fails to ascend and remains in the pelvis, known as a "pelvic kidney." Bilateral pelvic kidneys are an exceptionally rare anomaly, often with both kidneys positioned on the same side [1].

Congenital malformation uropathies are the third most common congenital anomaly, following cardiac and skeletal defects. These urinary system anomalies can affect the kidneys, ureters, bladder, or urethra in various ways. Renal developmental abnormalities, such as renal agenesis and ectopic kidneys, are among the possibilities, along with fusion anomalies [2].

Renal fusion anomalies are common congenital abnormalities of the urogenital system. They were first documented and classified by Wilmer in 1938, with McDonald and McClellan refining and expanding the classification in 1957 [2]. They are generally classified into partial fusion anomalies, such as crossed fused ectopia and horseshoe kidney, and complete fusion anomalies, like the pancake kidney (PK) [3].

The PK is a rare anomaly, accounting for only 2% of all cases of fused kidneys. Due to its characteristic ring- or doughnut-shaped structure, it is also known by various names, such as cake kidney, disc kidney, doughnut kidney, and shield kidney. In this condition, the renal pelvis faces anteriorly, the ureters do not cross, and the collecting systems of the kidneys are separate, with no communication between the 2 sides [1].

The PK may be associated with various urinary tract defects and anomalies in other organs. Patients with this condition may present with additional complications such as pelvic ureteric junction (UPJ) obstruction, recurrent urinary tract infections, and renal calculi [4]. The UPJ obstruction occurs when there is a blockage where the renal pelvis meets the ureter, preventing urine from flowing properly from the kidney to the bladder. If left untreated, it can lead to hydronephrosis and kidney damage [1].

This report presents a case of an infant diagnosed with pelvic PK accompanied by UPJ obstruction and an undescended right testis, an exceedingly rare combination of conditions. This report was prepared following the CaReL guidelines, with all references thoroughly reviewed for eligibility [5,6].

## Case presentation

**Fig. 1:**
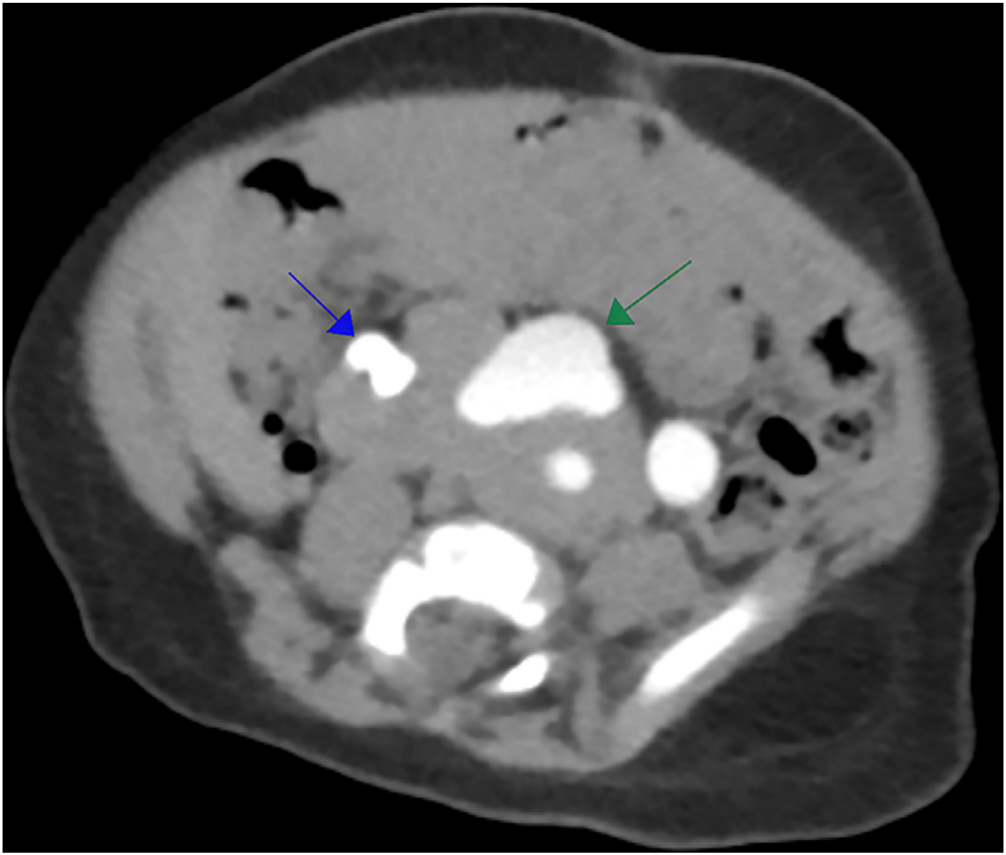
CT urography reveals the abnormal positioning of both kidneys. The right pelvicalyceal system is malrotated with no signs of dilation (blue arrow), whereas the left pelvicalyceal system is malrotated with moderate dilation, suggesting a PUJ obstruction (green arrow).

**Fig. 2:**
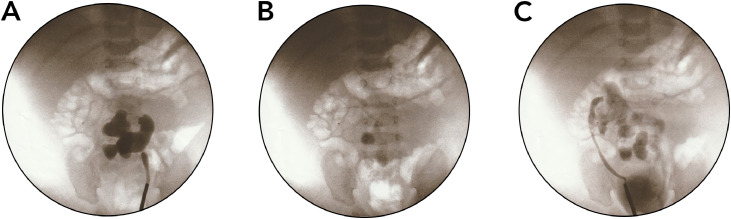
A retrograde pyelogram reveals: (A) A dilated pelvicalyceal system with mild PUJ stenosis on the left side. (B) Ten minutes after contrast injection, a nearly complete washout of the contrast is observed. (C) Twenty minutes after contrast injection, right-side retrograde pyelogram demonstrates a dilated pelvicalyceal system and fused kidneys.

A 2-month-old male infant was found to have an absent right testis during a routine check-up. Physical examination revealed an empty right hemiscrotum. An inguinoscrotal ultrasound (US) detected the right testis within the right inguinal canal, measuring 10 × 4.4 mm, with normal echotexture and vascularity. The scan also showed a mild hydrocele on the right side, while the left testis was normal. Further evaluation with the abdominal US revealed an unusual renal anomaly: both kidneys were located in the pelvis, positioned transversely, and displayed malrotation. Despite their ectopic location, the kidneys were normal in size and parenchymal thickness. The right kidney showed mild hydronephrosis, while the left had moderate hydronephrosis, with an anteroposterior diameter of 11 mm, indicating left UPJ obstruction. These findings suggested the presence of PK. Renal function tests revealed blood urea levels of 11.1 mg/dL (reference range: 11-39 mg/dL) and serum creatinine of 0.32 mg/dL (reference range: 0.17-0.42 mg/dL). The complete blood count was unremarkable. Imaging studies, including intravenous urography and computed tomography (CT) scans (Fig. 1), confirmed the anatomical abnormalities. Additionally, a MAG3 (mercaptoacetyltriglycine) renal scan further confirmed the diagnosis. The patient underwent left pyeloplasty. During the procedure, a retrograde pyelogram confirmed mild stenosis at the left PUJ, with contrast clearing within 10 minutes (Fig. 2). A left JJ stent was inserted, and a follow-up was planned. The stent was removed 6 weeks later. At a 3-month postoperative follow-up, ultrasound showed stable left hydronephrosis, and the infant remained asymptomatic. Additionally, a right-sided orchidopexy was performed through a right inguinal incision. The patient is now scheduled for follow-up visits every 6 months.

## Discussion

An ectopic kidney is a congenital condition in which the kidney is positioned abnormally, either lower, higher, or on the opposite side from its usual anatomical location. This anomaly occurs in approximately 0.1% of cases [1].

Horseshoe kidney is the most frequent form of ectopic kidney, with a prevalence of 0.1% in autopsy studies [1]. However, it accounts for 90% of all renal fusion anomalies [7]. In contrast, PK represents the rarest type of ectopic fused kidney, with an estimated occurrence of 1 in 65,000 to 375,000 individuals [8].

Generally, PK is more common in males, with a male-to-female ratio of 2.5:1. It is typically diagnosed between 30 and 60 years [1]. A review of 10 reported PK cases from the past decade revealed that 7 patients (70%) were male, and 3 (30%) were female, indicating a male predominance. The age at diagnosis ranged from 12 to 90 years (Table 1) [1,2,4,[9], [10], [11], [12], [13], [14], [15]]. In contrast, the current case was a 2-month-old male infant diagnosed with PK.

**Table 1:** Review of 10 cases of PK reported in the last decade.

**First author [Ref]**	**Year**	**Age (Y) /Sex**	**presentation**	**Kidney function**		**Diagnostic approaches**				**Diagnosis**
				**Urea**	**Creatinine**	**Dissection**	**X. ray**	**U/S**	**CT scan**	
Kanchan [1]	2016	23/M	Cadaver (Pronounced dead upon arrival after a traffic accident)	N/A	N/A	The right and left kidneys were out of place, and a pancake-shaped structure was seen in the right pelvic cavity.	N/A	N/A	N/A	Pelvic PK with a single ureter
Bakshi [2]	2020	12/F	Mild, dull pain in the lower abdomen accompanied by dysuria for 7 days.	Normal	Normal	N/A	Failed to demonstrate bilateral renal tissue shadows, with no evidence of calculus in the KUB region.	Normal intra-abdominal organs with empty bilateral renal fossae.	A round-shaped mass measuring approximately 9 cm in the pelvic cavity was ultimately identified as a pancake kidney.	Pelvic PK
Chaker [4]	2018	50/M	Renal colic for 2 months	N/A	N/A	Two abnormally rotated pelvic pancake kidneys fused at their outer edges.	N/A	Two discoid pelvic kidneys with dilation of the right renal pelvis and calyces.	Pancake kidneys with obstruction at the right ureteropelvic junction, along with multiple renal calculi.	Pelvic PK with UPJO, along with multiple renal calculi.
Horai [9]	2018	90/F	Cadaver (passed away due to pneumonia)	N/A	N/A	A PK with a single ureter was found in the left retroperitoneal space.	N/A	N/A	N/A	PK with a single ureter in the retroperitoneal space
Lomoro [10]	2018	14/F	Sudden onset of sharp pain in the right lower abdomen.	Normal	Normal	N/A	N/A	A fused pelvic kidney with lobulated contours, characteristic of a pancake kidney.	A large kidney mass with 2 fused lobes and a central isthmus of normal tissue was located in the hypogastric-left iliac region, anterior to L4-S1.	Pelvic PK
Pasquali [11]	2018	47/M	Presented for CT urography	Normal	Normal	N/A	N/A	N/A	A medially fused pelvic renal mass near the sacral promontory.	Pelvic PK
Singhal [12]	2018	18/M	Acute intestinal obstruction symptoms.	N/A	N/A	N/A	N/A	N/A	Bilateral ectopic kidneys in the lower abdomen, para-median between L4 and S2, fused at the upper and mid-poles across the midline.	Pelvic PK
Ghawanmeh [13]	2017	32/M	Lower limb pain for 2 months	N/A	Normal	N/A	N/A	N/A	A fused lobulated mass in the right abdomen (L2-L4) with uncrossed ureters opens separately into the bladder.	PK in the abdominal cavity
Kumar [14]	2017	65/M	A persistent, dull ache in the right flank and lower abdomen for 1 month	N/A	Normal	N/A	N/A	Two ectopic pelvic kidneys, 1 on each side, with a renal stone in the right kidney.	Fused ectopic renal structures in the pelvis with an anteriorly positioned hilum and a 2.5 cm stone in the right renal structure.	Pelvic PK associated with renal stone.
Musiienko [15]	2015	59/M	Rectosigmoid tumor causing obstruction.	N/A	N/A	Despite clear CT imaging, the right ureter was identified, but the left was not. Methylene blue confirmed no ureteric injury.	N/A	N/A	A fused pelvic kidney with separate collecting systems and ureters reaching normal vesicoureteric junctions.	Rectosigmoid carcinoma attached to a single pelvic PK.

CT, computed tomography; KUB, kidney, ureter, and bladder; N/A, nonavailable; PK, pancake kidney; U/S, ultrasound; UPJO, ureteropelvic junction obstruction; Y, year

The primary difference between a horseshoe kidney and a PK is the fusion level. A horseshoe kidney has minimal fusion, occurring only at the poles, while a PK shows full fusion, including the upper and lower poles and the hilum. This complete fusion results in the absence of an isthmus, with the kidneys appearing as a single overlapping mass. The PK is considerably rarer than a horseshoe kidney [1]. A horseshoe kidney can be found in both the abdomen and pelvis. In contrast, most previously reported cases of PK were located in the pelvic cavity and were drained by double ureters [11]. Ghawanmeh et al. [13] were the first to report a case of PK in the retroperitoneal space with double ureters. Kanchan et al. [1] described a case of PK with a single ureter located in the pelvic cavity during a medicolegal autopsy. In contrast, Horai et al. [9] reported a case of PK with a single ureter found in the left retroperitoneal space. However, the case described in this report involved PK with a double ureter in the pelvic cavity.

Although the exact mechanism behind renal fusion anomalies is not fully understood, several theories attempt to explain their developmental origins [1]. The human kidneys develop from the metanephros, with 2 masses of metanephric blastema initially located in the pelvis. As development progresses, the kidneys ascend and reach a lumbar position. This migration involves several complex movements, including ascent, lateral migration, axial deflection, and internal rotation [7]. Before these movements begin, the nephrogenic blastema is pushed between the umbilical arteries. Occasionally, fusion occurs during this pushing process, resulting in kidneys that fail to ascend and form a PK [7]. The genetic theory suggests that the Sonic Hedgehog gene plays a crucial role in kidney topography, and its disruption may lead to renal fusion anomalies [1].

Patients with PK often experience fever, painful urination, and lower abdominal discomfort due to urinary tract infections. Some individuals with fused pelvic kidneys maintain normal kidney function and remain asymptomatic [1]. The diagnosis of PK is typically discovered incidentally. Previously, excretory urography was used. However, it has now been replaced by US, multidetector computed tomography, CT urography, and radionuclide scanning, which provide more detailed imaging of the urinary system and renal vascular structure. The US is the primary method for diagnosing kidney abnormalities pre- and postnatally. The multidetector computed tomography with contrast enhancement is particularly effective for examining the anatomy of the urinary tract, including the kidney parenchyma and collecting systems [2]. The infant in this report was found to have an undescended right testis and was incidentally diagnosed with PK through ultrasound.

A PK may receive blood from branches of the abdominal aorta or multiple branches of the internal and external iliac arteries. Typically, the lower parts of the fused, ectopic kidneys are rotated more medially than the upper parts. The PK is generally located at the L3-L5 vertebral level and lies anterior to the major blood vessels. The parenchymatous or fibrous isthmus is situated where the inferior mesenteric artery originates from the aorta [2].

Commonly, PK is associated with various anomalies, including undescended testes, abnormal vas deferens, vaginal agenesis, unicornuate or bicornuate uterus, Fallot's tetralogy, spina bifida, sacral agenesis, caudal regression syndrome, and strabismus. It often exhibits a distorted and rotated collecting system. The shortened ureters can result in obstruction and urine stasis, leading to complications such as hydronephrosis, nephrolithiasis, vesicoureteral reflux, and recurrent urinary tract infections [2]. Chaker et al. [4] reported a case of PK with UPJ obstruction and multiple kidney stones, while Kumar et al. [14] described a case of PK complicated by renal stone formation. In the current case, the patient presented with PK associated with UPJ obstruction and an undescended right testis.

Evidence from available literature indicates that the likelihood of Wilms tumor in individuals with a horseshoe kidney is nearly double compared to those with normal renal anatomy. However, due to the limited number of reported cases of PK, the risk of developing Wilms tumor in these patients remains inconclusive [12].

The treatment of PK is mainly conservative for asymptomatic patients, with an emphasis on regular monitoring to prevent potential complications. Surgical treatment is considered only for symptomatic cases involving considerable renal dysfunction or obstruction [2]. In this case, the infant received a pyeloplasty to treat the UPJ obstruction and underwent right-sided orchidopexy for the undescended testis.

The PK is prone to injury during pelvic surgeries because of its atypical anatomy. This can pose challenges in surgeries involving the iliac vessels and abdominal aorta. Damage to the kidney’s parenchyma during surgery can lead to renal vascular injury, necrosis, infarction, and postoperative renal failure. Therefore, it is crucial for urologists and pelvic surgeons to be aware of the unique anatomy of the PK, particularly its blood vessels, to avoid unexpected severe bleeding. Preoperative vascular assessments may be necessary to prevent such complications due to the presence of accessory renal arteries [1].

## Conclusion

The PK may coexist with conditions such as UPJ obstruction and undescended testes. It can be managed conservatively if asymptomatic, with monitoring to prevent complications. This rare association warrants further investigation to elucidate potential shared embryologic mechanisms.

## Patient consent

Consent has been taken from the parents of the patient.
